# Self-care practices among heart failure patients: A cross-sectional study in Saudi population

**DOI:** 10.15537/smj.2023.44.3.20220799

**Published:** 2023-03

**Authors:** Khalid A. Aljohani

**Affiliations:** *From the Department of Community Health Nursing, College of Nursing, Taibah University, Al Madinah al Munawarah, Kingdom of Saudi Arabia.*

**Keywords:** self-care, heart failure, heart failure index, Saudi Arabia

## Abstract

**Objectives:**

To describe self-care practices among Saudi heart failure (HF) patients and identify sociodemographic characteristics contributing to self-care practices.

**Methods:**

A cross-sectional study utilizing the Arabic-language version of the revised Self-Care of Heart Failure Index (SCHFI), version 7-2. A convenience sample of 245 people treated for HF at a tertiary heart center in the Kingdom of Saudi Arabia were recruited from June to August 2020.

**Results:**

Statistical descriptions of SCHFI showed that confidence level was 84%, maintenance level was 67.5%, and monitoring level was 67.2%. Females’ HF management (*p*=0.023) and confidence (*p*=0.002) were significantly higher than male participants. In addition, education level and employment status had a significant effect on HF monitoring with a *p*-value of 0.006 for the 4 employment categories (F=[3,241]=4.06, *p*=0.008, h^2^=0.048). The effect size was small to medium for education level and employment status in the abovementioned results. Confidence significantly contributed to explaining all self-care sub-scale scores. Independent variables significantly predicted monitoring subscale scores (R2=0.082, F=[7,237]=3.027, *p*=0.005).

**Conclusion:**

Self-care practices in this study showed higher scores than those reported in international studies. Further studies are warranted to explore everyday self-care needs and challenges among HF patients.

**C**ardiovascular diseases (CVD) account for approximately 18 million deaths per year worldwide, with an increase of 14% since 2006, an estimated prevalence of 6.2 million heart failure (HF) cases in the United States.^1^ Moreover, estimated HF mortality with a 12-month incidence in developing regions was highest in Africa (34%), followed by India (23%), and 9% in the Middle East.^2^ According to AlHabeeb et al,^3^ there are 1.35 million people with HF in Egypt, Saudi Arabia, and the United Arab Emirates. The estimated HF population in Saudi Arabia is 320,933 people with a yearly healthcare expenditure of more than one Billion American dollars ($1.045B), expended mainly on hospitalization. Patient hospitalization costs ranged between $3,671-16,447 on yearly basis.^4^ The burden of HF is not limited to the healthcare system and patients financial lose, HF consequences are impacting the entire society in terms of economic burden, minimized citizens’ quality of life, decreased productions, overwhelming family members and carers’ obligations, and patients’ inability to care for themselves.^4-7^

Heart failure is a progressive condition that may result in disability. Aging, expansion of non-communicable diseases, and sedentary lifestyles are factors contributing to HF being a leading epidemic with severe negative health, economic, and social impacts.^8,9^ Numerous strategies have been implemented internationally to minimize the impact of HF. The British Heart Foundation adapted the community HF specialist nurse, which resulted in a 35% reduction in the re-admission rate and an estimated cost savings of 169,000 pound (£) per 1000 patients.^9^

In specific, international initiatives have mainly targeted the improvement of self-care practices/behavior among people diagnosed with HF, with the assistance of a multidisciplinary healthcare team.^10,11^ Proper self-care practices had a positive influence on enhancing health-related quality of life (HRQoL) and clinical outcomes. Despite these outcomes, patients may still do not follow the recommended self-care practices and do not get proper attention from healthcare teams in the continuum.^12,13^

Self-care practices are influenced by external as well as individual’s sociodemographic factors. Socio-demographics such as age, gender, education level, income level, and employment status were explored in several studies. However, the outcomes of these studies were inconsistent with each other in terms, which factor was positively correlated to higher self-care practices.^7,14-19^

From a national standpoint, Saudi Arabia had no standardized HF management protocols until 2019. The recent Saudi Heart Association (SHA) guidelines for HF management were developed to bridge this gap in Saudi healthcare organizations.^10^ Reviewing the guidelines revealed minimal identification of self-care interventions, which appeared only as a brief recommendation in the section on “Multidisciplinary team management.” Moreover, exploring factors contributing to HF self-care was cited in few Saudi studies compared to international HF literature.^14,20^ Elkhateeb et al^20^ explored the effect of education level on hospital readmission and mortality rates in King Abdullah Medical City, Riyadh, Saudi Arabia, for 167 patients monitored in a 12-month follow-up. The study did not identify significant differences between highly educated participants and those with lower levels. Regarding medication adherence, Raffaah et al^14^ found that optimum adherence was evident in 7.3% of the study sample. In addition, researchers identified sociodemographic factors (single, unemployed, and low income) as predictors of low medication adherence. A structured program to improve HF patients’ outcomes was tested in a randomized control trial and found that those who received the intervention were 95% less likely to be readmitted during the 4-month study period.^21^

The scarcity of HF nursing studies in Saudi Arabia may contribute to the limited number of studies devoted to HF self-care, as nurses are more concerned with patient-centered care and advocacy.^22^ The position statement of the American Association of Heart Failure Nurses (AAHFN) recommended that nurses should work to understand the factors contributing to enhanced HF self-care practices.

In general, international self-care recommendations could not be transformed into Saudi context due to difference in individual and cultural characteristics. Therefore, the aims of this study were to describe self-care practices among Saudi HF patients and determine the sociodemographic factors that contribute to self-care practices. The outcome of this study will inform healthcare team as well as contribute to HF self-care body of knowledge in Saudi Arabia.

## Methods

A cross-sectional descriptive correlational design was applied. The study was carried out in Al-Madinah Cardiac Center, Al-Madinah Al-Munawarah, Saudi Arabia, one of 25 heart care centers and departments across the Kingdom. The study site was selected as a representative of other centers in Saudi Arabia due to its characteristics in terms of patients’ number, case variety, and advanced cardiac services. It is a 100-bed facility with an estimated 30,000 registered patients and 80,000 annual outpatient department (OPD) visits. The study was carried out in the OPD.

A convenience sampling technique was used to recruit patients with HF who were attending their follow-up consultation appointments at Al-Madinah Cardiac Center, Al-Madinah Al-Munawarah, Saudi Arabia. The sampling approach was adapted from an earlier study in Jordan.^7^ The study included patients who: I) were diagnosed with HF; II) had a reduced ejection fraction (EF) of ≤45%; III) were at least 18 years old; IV) were able to agree to voluntary participation without mental or medical limitations and contraindications; and V) were registered in the cardiac center outpatient clinics. Inclusion criteria were checked through electronic medical records. People who were unable to communicate properly in Arabic or English were excluded. The sample size (n=160) was calculated via G*Power (α=0.05; effect size [f^2^]=0.15, power [1-β]=0.95 and IV=7).^15^ However, 270 questionnaires were distributed to compensate for potential invalid or incomplete responses.

The study protocol was approved by Al-Madinah Cardiac Center Institutional Review Board (no.: 2020R29). The study conforms with the principles outlined in the Declaration of Helsinki. The Strengthening the Reporting of Observational studies in Epidemiology (STROBE) checklist was utilized to enhance study methodology.

The study utilized the Arabic version of the revised Self-Care of Heart Failure Index (SCHFI V.7-2), which is available in the public domain.^23^ The instrument identifies the outcome variable (self-care) by exploring 3 concepts: I) self-care (10 items); II) self-care maintenance (9 items plus 2 on patients’ ability to detect symptoms); and III) symptom perception and self-care management (8 items). Because the SCHFI is a multidimensional instrument, the reliability coefficient (RC) is a better fit than the popular Cronbach’s alpha reliability measure.^24^ Reliability coefficients of the original instrument were ≥70.^25^ For the translated Arabic version, they were ≥87.^26^

Responses are ranked on a Likert scale. The scoring system provided by the instrument’s owner standardizes responses to 100 (a higher score indicates more effective self-care practices). The instrument has shown acceptable psychometric properties and has been translated into various languages, including Arabic.^7,26,27^

Data were collected during the period from June 2020 to August 2020. The cardiac clinic nurse or the outpatient clinic nursing supervisor invited qualified potential participants upon their attendance for their clinic visit. The nursing supervisor referred those who were willing to participate to the data collection team (2 nursing interns who were trained to carry out data collection interview). Data collection was carried out in the clinic waiting area. Potential participants were introduced to the study verbally and given the study information sheet that included the consent form agreeing to participate. Some who did not wish to sign were included, considering their responses to the study questionnaire as consent to participate. Participants were offered the option to record their responses through the data collection team, who documented them in the study questionnaire using paper and pencil. Completed questionnaires were returned weekly to the researcher. Duration of data collection for each participant was between 8-12 minutes.

### Statistical analysis

Descriptive and correlational statistical analyses were carried out using the Statistical Package for the Social Sciences, version 20.0 (IBM Corp., Armonk, NY, USA). Patient’s characteristics and socio-demographic variables were tested to explore self-care practices. Mean and standard deviation (SD) of continuous variables and frequencies for categorical variables were used to describe the participants’ characteristics and the instrument’s subscales. Independent sample t-tests and ANOVA were used to explore significant differences in self-care practices among various participants’ characteristics. A multiple linear regression analysis was carried out to identify factors associated with self-care practices including confidence (loaded as subscale in the SCHFI).

## Results

A sample of 245 HF participants responded to the survey, with an overall response rate of 90.7%. Those who did not respond were mainly concerned on the time that the questionnaire may take. Sociodemographic characteristics (Table 1) reveal that the majority were male (70%) and that approximately 27% had no formal education, while 50% had general education (primary-elementary and secondary schools). The participants were 56 years old on average, had 3.6 years of disease duration, had one hospital admission in the previous year, and had 5 family members living with them.

**Table 1:** - Sociodemographic characteristics of the participants.

Characteristics	n (%)
***Gender***	
Male	172 (70.2)
Female	73 (29.8)
***Education***	
No education	67 (27.3)
General education	123 (50.2)
Graduate	55 (22.4)
***Employment***	
Not employed	102 (41.6)
Retired	84 (34.3)
Government	38 (15.5)
private	21 (8.6)
***Marital status***	
Single/widow	50±20.4
Married	195±79.6
Age, mean±SD	56.51±9.70
Disease duration since diagnosis, mean±SD	3.60±4.43
Hospital admissions within the last year, mean±SD	1.18±1.52
Family size, mean±SD	4.86±3.38

Values are presented as numbers and precentages or mean ± standard deviation (SD).

Table 2 shows that the confidence scale was highest (84%), while maintenance scale ranked 67.5% and monitoring scale ranked 67.2%. Highest and lowest items’ means for each scale are shown in Table 2. The 2 extra items loaded onto the monitoring scale; “how quickly did you recognize that you had symptoms?” and “how quickly did you know that the symptom was due to HF?” had low mean scores (2.95±1.67 and 2.24±1.88).

**Table 2:** - Descriptive statistics of self-care of heart failure index.

Scale and upper-lower scored items	Mean±SD	Converted to 100*
***Maintenance sub-scale***	3.375±0.647	67.5%
Yearly flu vaccine	2.24±1.50	
Not missing doses	4.41±0.982	
***Monitoring sub-scale***	3.36±0.721	67.2%
Recorded symptoms	1.82±1.33	
Noticed tiredness	4.09±1.09	
Recognize symptoms	2.95±1.67	
Know symptoms	2.24±1.88	
Management sub-scale	3.75±0.569	75.0%
Call for guidance	2.70±1.69	
Take medicine	4.63±0.904	
***Confidence sub-scale***	4.19±0.695	83.8%
Evaluate remedy	4.00±1.11	
Follow treatment plan	4.53±0.738	

Values are presented as mean ± standard deviation (SD).

* Mean/highest scale number (5) × 100

Table 3 provides comparisons of means among participants’ sociodemographic data. Female participants scored higher in management and confidence than males. The mean management scale for females was significantly higher than that of males (t[243]=2.29; d=0.31; *p*=0.023). Similarly, the mean on the confidence scale for females was significantly higher than that of males (t[243]=3.09; d=0.42; *p*=0.002). Level of education had a significant effect on HF management with a *p*-value of 0.006 for the 3 categories (F=[2,242]=5.18; *p*=0.006; η^2^=0.041). In addition, employment status had a significant effect on HF monitoring with a *p*-value of 0.008 for the 4 employment categories (F=[3,241]=4.06; *p*=0.008; η^2^=0.048). The effect size was small to medium for education level and employment status in the aforementioned results.

**Table 3:** - Differences in self-care practices.

Variables	Maintenance	Monitoring	Management	Confidence
***Gender***				
Male	3.377±0.638	3.332±0.725	3.70±0.651	4.10±0.724
Female	3.372±0.672	3.43±0.710	3.88±0.572	4.40±0.573
T-test	t(243)=0.052, *p*=0.958	t(243)=1.03, *p*=0.302	t(243)=2.29, *p*=0.023*	t(243)=3.09, *p*=0.002*
***Education***				
No education	3.34±0.59	3.24±0.73	3.79±0.57	4.26±0.64
General education†	3.44±0.64	3.44±0.67	3.83±0.53	4.15±0.71
Graduate	3.79±0.57	3.32±0.80	3.54±0.59	4.20±0.72
ANOVA	F=(2,242)=1.66, *p*=0.191	F=(2,242)=1.82, *p*=0.164	F=(2,242)=5.18, *p*=0.006*	F=(2,242)=0.502, *p*=0.606
***Employment***				
Not employed	3.36±0.59	3.38±0.65	3.81±0.57	4.25±0.67
Retired	3.48±0.65	3.46±0.73	3.79±0.58	4.21±0.67
Government	3.33±0.67	3.36±0.73	3.63±0.55	4.10±0.77
Private	3.06±0.67	2.86±0.77	3.59±0.45	4.00±0.71
ANOVA	F=(3,241)=2.47, *p*=0.062	F=(3,241)=4.06, *p*=0.008*	F=(3,241)=1.60, *p*=0.189	F=(3,241)=0.978, *p*=0.404
***Marital status***				
Single	3.23±0.63	3.37±0.67	3.77±0.55	4.23±0.79
Married	3.41±0.64	3.36±0.73	3.75±0.57	4.18±0.66
T-test	t(243)=1.74, *p*=0.082	t(243)=0.105, *p*=0.916	t(243)=0.269, *p*=0.789	t(243)=0.461, *p*=0.645

Values are presented as mean ± standard deviation (SD). ^*^Significance level at 0.05. ^†^General education include primary, secondary, and high school education. ANOVA: analysis of variance

Multiple linear regression analysis revealed some significant factors contributing to self-care practices. The total score was not significantly explained by ID variables despite it was close to the significance level alpha (*p*=0.072). However, ID variables significantly predicted monitoring subscale scores (R^2^=0.082; F[7,237]=3.027; *p*=0.005), details are provided in Table 4. Confidence level significantly predicted maintenance subscale scores (β=0.364; R^2^=0.153; F[1,234]=43.7; *p*=0.000); monitoring subscale scores (β=0.530; R^2^=0.260; F[1,234]=85.6; *p*=0.000); and management subscale scores (β=0.384; R^2^=0.219; F[1,234]=68.31; *p*=0.000).

**Table 4:** - Factors contributing to Self-care practices.

Variables	β	R^2^	F	*P*-values
***Self-care behaviors (total score)***				
Gender	0.093			
Age	2.35			
Disease duration in years	0.005			
Hospital admission(s)	0.026	0.053	1.891	0.072
Number of family members	0.006			
Education	0.131			
Employment	0.233			
***Maintenance***				
Gender	0.008			
Age	0.004			
Disease duration in years	0.002			
Hospital admission(s)	0.005	0.021	0.736	0.640
Number of family members	0.002			
Education	0.117			
Employment	0.160			
Confidence	1.84	0.153	43.7	0.000
***Monitoring***				
Gender	0.098			
Age	0.004			
Disease duration in years	0.007			
Hospital admission(s)	0.085	0.082	3.027	0.005
Number of family members	0.014			
Education	0.27			
Employment	0.348			
Confidence	1.141	0.260	85.6	0.000
***Managemen*** t				
Gender	0.173			
Age	0.001			
Disease duration in years	0.005			
Hospital admission(s)	0.001	0.041	1.437	0.191
Number of family members	0.007			
Education	0.038			
Employment	0.193			
Confidence	2.148	0.219	68.314	0.000

## Discussion

Participants sociodemographic characteristics were similar to earlier national studies in the field of cardiovascular healthcare research. For instance, the mean age of 56 years and male dominance (70%) in this study were within the range of earlier Saudi studies.^14,20^ Such similarities could be attributed to the HF population, which is also presented in international studies.^5,28^ Therefore, the convenience-sampling approach did not change the target population characteristics.

In general, the study result accomplished the main 2 aims that are describing self-care practices among Saudi HF patients and determining the sociodemographic factors that contribute to self-care practices. From the first study aim stand, the self-care maintenance sub-scale scored 67.5% and monitoring sub-scale scored 67.2%, which are higher than those reported in earlier international studies. For instance, the maintenance subscale was 48% in China, 62% in the United States of America, and 53% in Jordan.^29-31^ The lower threshold of accepted self-care practices was ≥70.^13^ In specific, continuous healthcare delivery capabilities may keep HF patients under close monitoring. This explanation was supported by the significant explanation of the hospital admissions to 85% of the monitoring scale.

However, this may not reflect a high patient engagement approach as much as the healthcare system’s monitoring capabilities. Specifically, checking the 2 extra items in the monitoring sub-scale; “How quickly did you recognize that you had symptoms” and “How quickly did you know that the symptom was due to heart failure” revealed low scores. Therefore, participants were unable to identify or recognize symptoms because they were dependent on the healthcare system regarding disease-related matters.

Self-care management (75%) was also higher than that of earlier international studies, where the reported scores ranged between 45-68%.^5,29,30^ Following the treatment plan was the highest item on the sub-scale (4.53±0.738), contrasting with earlier poor medication adherence reported by an earlier national study.^14^ Therefore, future studies should use recommended international approaches to measure HF patients’ medication adherence, such as continuous data monitoring.^32^

From the second study aim stand to determine the factors contributing to self-care practices; interestingly management score was higher in female participants, indicating a higher application of the treatment plan. Gender was not a factor in earlier international studies.^6,7^ In this study, education level had a significant effect on HF management. Participants with a general education (public education) scored higher than other groups. Such significance was not evident in the earlier national study.^14^ International literature suggests that health literacy contributes to medication adherence.^33^ In general, interventions to enhance patients’ HF literacy are crucial for building their self-care capabilities.^26^

Explaining independent variables’ contribution to self-care through multiple linear regression analysis revealed some significant factors but not to the total self-care practices score. The total score was not significantly explained by IV’s despite it being close to the significance level alpha value of 0.050. However, IV’s significantly predicted 8.2% of the monitoring subscale scores. Similar to previous international studies, confidence significantly predicted the Self-Care of Heart Failure Index subscales.^34,35^ In this study, confidence significantly predicted 15.3% of the maintenance, 26% of the monitoring, and 21.9% of the management subscale scores. However, international studies showed higher prediction score. For example, in Lebanon, confidence predicted 26% of the maintenance, 34% of the monitoring, and 24% of management.^34^

Assessing HF self-care practices in the Saudi population using the Arabic version of the revised Self-Care of Heart Failure Index (V.7-2) and other widely used international instruments is crucial for comparisons between national and international outcomes. Moreover, understanding factors and approaches to engage HF patients’ participation in their healthcare practices identified as self-care or self-management is a cornerstone for successful healthcare interventions;^36^ thus, this study may also contribute to a better understanding of such factors among Saudi HF patients.

### Study limitations

The generalizability is limited due to cross-sectional design, convenience sampling, and single data collection location. On the other side, these factors facilitate smooth HF participants’ flow to the study location during the study period. In addition, comparisons with national and international studies revealed acceptable compatibility, in terms of gender and age representation. This does not preclude the need for further studies with more representative sample utilizing a longitudinal study design. From a positive perspective, the study is a positive step towards addressing HF patients’ self-care practices and factors that may enhance their care management and quality of life.

In conclusion, this study shed light on HF patient self-care practices in one cardiac healthcare organization in Saudi Arabia. Participants showed sub-optimal HF self-care practices as well as the confidence to practice self-care activities. Female gender, education status, and hospital admissions were correlated with higher self-care. Confidence was a major predictor for all self-care sub-scales. Therefore, focusing on building confidence among HF patients could be an area of a priority in healthcare interventions.
